# IRAK1/4-Targeted Anti-Inflammatory Action of Caffeic Acid

**DOI:** 10.1155/2013/518183

**Published:** 2013-11-27

**Authors:** Woo Seok Yang, Deok Jeong, Young-Su Yi, Jae Gwang Park, Hyohyun Seo, Sang Hyun Moh, Sungyoul Hong, Jae Youl Cho

**Affiliations:** ^1^Department of Genetic Engineering, Sungkyunkwan University, Suwon 440-746, Republic of Korea; ^2^Anti-aging Research Institute, BIO-FD&C Co., Ltd, Incheon 406-840, Republic of Korea

## Abstract

Caffeic acid (CA) is a phenolic compound that is frequently present in fruits, grains, and dietary supplements. Although CA has been reported to display various biological activities such as anti-inflammatory, anti-cancer, anti-viral, and anti-oxidative effects, the action mechanism of CA is not yet fully elucidated. In this study, the anti-inflammatory action mechanism of CA was examined in lipopolysaccharide (LPS) treated macrophages (RAW264.7 cells) and HCl/EtOH-induced gastritis. CA was found to diminish nitric oxide (NO) and prostaglandin E_2_ (PGE_2_) production in LPS-stimulated RAW264.7 cells. Additionally, mRNA levels of tumor necrosis factor (TNF)-**α**, cyclooxygenase (COX)-2, and inducible NO synthase (iNOS) were downregulated by CA. CA also strongly suppressed the nuclear translocation of AP-1 family proteins and the related upstream signaling cascade composed of interleukin-1 receptor-associated kinase 1 (IRAK1), IRAK4, TGF-**β**-activated kinase 1 (TAK1), mitogen-activated protein kinase kinase 4/7 (MKK4/7), and c-Jun N-terminal kinase (JNK). In a direct kinase assay, CA was revealed to directly inhibit IRAK1 and IRAK4. CA also ameliorated HCl/EtOH-induced gastric symptoms via the suppression of JNK, IRAK1, and IRAK4. Therefore, our data strongly suggest that CA acts as an anti-inflammatory drug by directly suppressing IRAK1 and IRAK4.

## 1. Introduction

Upon infection by foreign pathogens, innate immune cells, which include macrophages, keratinocytes, and Langerhans cells, are activated via pattern recognition receptors such as the toll-like receptors (TLRs). Once activated, these cells can phagocytose materials from pathogens and infected cells and subsequently produce various inflammatory mediators such as interleukin (IL)-1, IL-6, tumor necrosis factor (TNF)-*α*, and the mediators nitric oxide (NO) and prostaglandin E_2_ (PGE_2_) that can stimulate other immune cells [1, 2]. Numerous intracellular signaling cascades, including non-receptor protein tyrosine kinases, phosphoinositide 3-kinase (PI3K), phosphoinositide-dependent kinase 1 (PDK1), and mitogen-activated protein kinases (MAPK) that are required for the translocation of transcription factors such as nuclear factor (NF)-*κ*B and activator protein (AP)-1, are involved in these processes [3, 4]. Consequently, inflammatory cells can express numerous inflammatory genes that encode proinflammatory cytokines, inducible NO synthase (iNOS) for NO release, and cyclooxygenase (COX)-2 for prostaglandin E_2_ (PGE_2_) production [5–8]. The excessive activation of inflammatory cells is known to generate an immunopathological environment in which tissues or organs can be damaged, thus leading to various inflammatory diseases such as arthritis, cancer, and atherosclerosis [9–11]. Therefore, current research has focused on the development of therapeutic remedies that can regulate cellular and molecular inflammatory responses.

Caffeic acid (CA) is a representative phenolic compound that is found in many different natural resources, such as fruits, vegetables, and herbs [12]. CA has been known to possess numerous biological activities such as anti-oxidative, anti-cancer, anti-viral, anti-inflammatory, and anti-diabetic effects [12–14]. It is not yet fully understood how this compound can display such a wide spectrum of biological activities. However, several reports have indicated that this compound can simultaneously suppress the activation of various transcription factors such as NFAT, NF-*κ*B, and AP-1 [15, 16]. Although some reports have suggested Fyn, 5-lipoxygenase, matrix metalloproteinase, and ectonucleotidase as the pharmacological targets of CA and its chemical derivates, including CA phenethyl ester [17–20], few have reported on the direct target of CA in the regulation of transcription factor activation. Therefore, in this study, we aimed to explore the anti-inflammatory potential of CA and the target enzyme(s) that contribute to CA-associated anti-inflammatory activity in LPS-activated macrophages and an HCl/EtOH-treated gastritis model.

## 2. Materials and Methods

### 2.1. Materials

CA (purity: 95%), 3-(4,5-dimethylthiazol-2-yl)-2,5-diphenyltetrazolium bromide (MTT), phorbol 12-myristate 13-acetate (PMA), and lipopolysaccharide (LPS; *E. coli* 0111:B4) were purchased from Sigma Chemical Co. (St. Louis, MO, USA). SP600125 was obtained from Calbiochem (La Jolla, CA, USA). Methanol extracts prepared from *Echinacea purpurea* (Ep-ME: FBM094-078), *Nyctanthes arbortristis* (Na-ME: FBM015-052), and *Vernonia cinerea* (Vc-ME: FBM028-094), all of which are known to contain CA [21–23], were purchased from the Plant Extract Bank at the Plant Diversity Research Center (http://extract.pdrc.re.kr/extract/f.htm, Daejeon, Republic of Korea). Luciferase constructs that contained promoters sensitive to NF-*κ*B, CREB, and AP-1 were gifts from Profs. Hae Young Chung (Pusan National University, Pusan, Republic of Korea) and Man Hee Rhee (Kyungpook National University, Daegu, Republic of Korea). Enzyme immunoassay (EIA) kits for determining PGE_2_ concentrations were purchased from Amersham (Little Chalfont, Buckinghamshire, UK). Fetal bovine serum and RPMI 1640 were obtained from Gibco (Grand Island, NY, USA). The RAW264.7 murine macrophage cell line and the HEK293 human embryonic kidney cell line were purchased from the ATCC (Rockville, MD, USA). All other chemicals were of analytical grade and were obtained from Sigma. Phosphospecific or total antibodies against p65, c-Jun, c-Fos, FRA-1, JNK, MKK4/7, TAK1, IRAK1, IRAK4, lamin A/C, and *β*-actin were obtained from Cell Signaling (Beverly, MA, USA).

### 2.2. Animals and Ethics Statement

Male ICR mice (6–8 weeks old, 17–21 g) were obtained from Daehan Biolink (Chungbuk, Republic of Korea). The mice were maintained in plastic cages in a temperature-controlled (24  ±  1°C) room on a 12-h light/dark cycle and given free access to pelleted food (Samyang, Daejeon, Republic of Korea) and water. Each mouse was used only once during the experiment. The use of animals according to the Guiding Principles in the Care and Use of Animals of the American Physiology Society and was approved by the Institutional Animal Care and Use Committee of Sungkyunkwan University (Approval ID: 201202018). Every effort was made to minimize the number of animals used and their suffering. Sacrifice of the mice was performed under isoflurane anesthesia.

### 2.3. Cell Culture

RAW264.7 and HEK293 cells were cultured in DMEM or RPMI 1640 medium that was supplemented with 10% heat-inactivated fetal bovine serum (FBS; Gibco, Grand Island, NY, USA), glutamine, and antibiotics (penicillin and streptomycin) (Gibco, Grand Island, NY, USA) at 37°C and in 5% CO_2_. For each experiment, cells were detached with a cell scraper. The proportion of dead cells at the cell density used for the experiments (2 × 10^6^ cells/mL) was less than 1%, as measured by Trypan blue dye exclusion.

### 2.4. Cell Viability Test

After an 18-h preincubation of RAW264.7 cells (1 × 10^6^ cells/mL), CA (0 to 400 *μ*M) was added to the cells, which were subsequently incubated for 24 h. The cytotoxic effect of CA was then evaluated with a conventional MTT assay as reported previously [24, 25]. At 3 h prior to culture termination, 10 *μ*L of MTT solution (10 mg/mL in phosphate buffered-saline, pH 7.4) was added to each well, and the cells were continuously cultured until termination of the experiment. The incubation was halted by the addition of 15% sodium dodecyl sulfate (SDS) to each well to solubilize the formazan [26]. Absorbance at 570 nm (OD_570–630_) was measured on a SpectraMax 250 microplate reader (Molecular Devices, Sunnyvale, CA, USA).

### 2.5. Determination of NO and PGE_2_ Levels

After an 18-h preincubation of RAW264.7 cells (1 × 10^6^ cells/mL), the cells were pretreated with CA (0 to 400 *μ*M) for 30 min and were further incubated with LPS (1 *μ*g/mL) for 24 h. The inhibitory effects of CA on NO and PGE_2_ production were determined by analyzing NO and PGE_2_ levels with Griess reagent and enzyme-linked immunoassay (EIA) kits, respectively, as previously described [27, 28].

### 2.6. Semiquantitative Reverse Transcriptase (RT) and Real-Time Polymerase Chain Reaction (PCR) Analysis of mRNA Levels

To determine the mRNA expression levels of various cytokine genes, total RNA was isolated from LPS-activated RAW264.7 cells with TRIzol Reagent (Gibco, Grand Island, NY, USA), according to the manufacturer's instructions. Briefly, RAW264.7 cells were pretreated with CA (100 to 400 *μ*M) for an additional 30 min, followed by a 6-h treatment with LPS (1 *μ*g/mL). Total RNA was stored at −70°C until use. Semiquantitative RT reactions were performed as previously reported [29, 30]. Quantification of mRNA was performed by real-time RT-PCR with SYBR Premix Ex Taq, according to the manufacturer's instructions (Takara Bio. Inc., Shiga, Japan), and the reactions were performed on a real-time thermal cycler (BioRad, Hercules, CA, USA) as previously reported [31, 32]. The results were expressed as the ratio of the optimal density relative to GAPDH. The primers used (Bioneer, Daejeon, Republic of Korea) are listed in Table 1.

### 2.7. Luciferase Reporter Gene Activity Assay

HEK293 cells (1 × 10^6^ cells/mL) in a 12-well plate were transfected with 1 *μ*g of plasmid that contained NF-*κ*B-Luc, CREB-Luc, or AP-1-Luc along with *β*-galactosidase via the calcium phosphate method, according to the manufacturer's protocol [33]. The cells were used for experiments at 48 h after transfection. Luciferase assays were performed with the Luciferase Assay System (Promega, Fitchburg, WI, USA) as previously reported [34].

### 2.8. Preparation of Cell Lysates and Nuclear Fractions, Immunoblotting, and Immunoprecipitation

Mouse stomach tissues or RAW264.7 cells (5 × 10^6^ cells/mL) were washed three times in cold PBS with 1 mM sodium orthovanadate and were lysed by a Tissuemizer (Qiagen, Germantown, MD, USA) or a sonicator (Thermo Fisher Scientific, Waltham, MA, USA), respectively in lysis buffer (20 mM Tris-HCl, pH 7.4, 2 mM EDTA, 2 mM ethyleneglycotetraacetic acid, 50 mM *β*-glycerophosphate, 1 mM sodium orthovanadate, 1 mM dithiothreitol, 1% Triton X-100, 10% glycerol, 10 *μ*g/mL of aprotinin, 10 *μ*g/mL of pepstatin, 1 mM of benzimide, and 2 mM PMSF) for 30 min with rotation at 4°C. The lysates were clarified by centrifugation at 16,000 ×g for 10 min at 4°C and stored at −20°C until being needed.

Nuclear lysates were prepared in a three-step procedure [35]. After treatment, the cells were collected with a rubber policeman, washed with PBS, and lysed in 500 *μ*L of lysis buffer with 50 mM KCl, 0.5% Nonidet P-40, 25 mM HEPES (pH 7.8), 1 mM phenylmethylsulfonyl fluoride, 10 *μ*g/mL of leupeptin, 20 *μ*g/mL of aprotinin, and 100 *μ*M 1,4-dithiothreitol (DTT) on ice for 4 min. Next, the cell lysates were centrifuged at 19,326 ×g for 1 min in a microcentrifuge. In the second step, the nuclear fraction pellet was washed once in wash buffer (lysis buffer without Nonidet P-40). In the final step, the nuclei were treated with an extraction buffer that contained 500 mM KCl and 10% glycerol in addition to the other reagents in the lysis buffer. The nuclei/extraction buffer mixtures were frozen at −80°C, thawed on ice, and centrifuged at 19,326 ×g for 5 min. The supernatants were collected as the nuclear extract. Soluble cell lysates were immunoblotted, and the protein levels were visualized as previously reported [36]. For immunoprecipitation, cell lysates with equal amounts of protein (500 *μ*g) from RAW264.7 cells (1 × 10^7^ cells/mL) that had been treated with or without LPS (1 *μ*g/mL) for 2.5 min were precleared with 10 *μ*L of protein A-coupled Sepharose beads (50% v/v; Amersham, Little Chalfont, Buckinghamshire, UK) for 1 h at 4°C. Precleared samples were incubated with 5 *μ*L of anti-IRAK4 antibody overnight at 4°C. The immune complexes were mixed with 10 *μ*L of protein A-coupled Sepharose beads (50% v/v) and rotated for 3 h at 4°C.

### 2.9. Enzyme Assay

The kinase profiler service from Millipore (Billerica, MA, USA) was used to evaluate the inhibition of IRAK1 and IRAK4 activity with purified enzymes. Purified enzymes (1–5 mU) were incubated with reaction buffer in a final reaction volume of 25 *μ*L. The reactions were initiated by the addition of MgATP. After a 40-min incubation at room temperature, the reactions were stopped by the addition of 5 mL of 3% phosphoric acid. Ten microliters of each reaction mixture was spotted onto a P30 filtermat and washed three times for 5 min each with 75 mM phosphoric acid and once with methanol prior to drying and scintillation counting.

### 2.10. EtOH/HCl-Induced Gastritis

Stomach inflammation was induced with EtOH/HCl according to a previously published method [37]. Fasted ICR mice were orally treated with CA (100 and 200 mg/kg) or ranitidine (40 mg/kg) twice daily for three days. Thirty minutes after the final injection, 400 *μ*L of 150 mM HCl in 60% ethanol was administered orally. Each animal was anaesthetized and sacrificed with an overdose of urethane at 1 h after the administration of the necrotizing agents. The stomachs were excised and gently rinsed under running tap water. After opening each stomach along the greater curvature and spreading it out on a board, the area (mm^2^) of the mucosal erosive lesions was measured with a pixel-counter by a technician blinded to the treatment conditions.

### 2.11. Statistical Analysis

**Figure 1:**
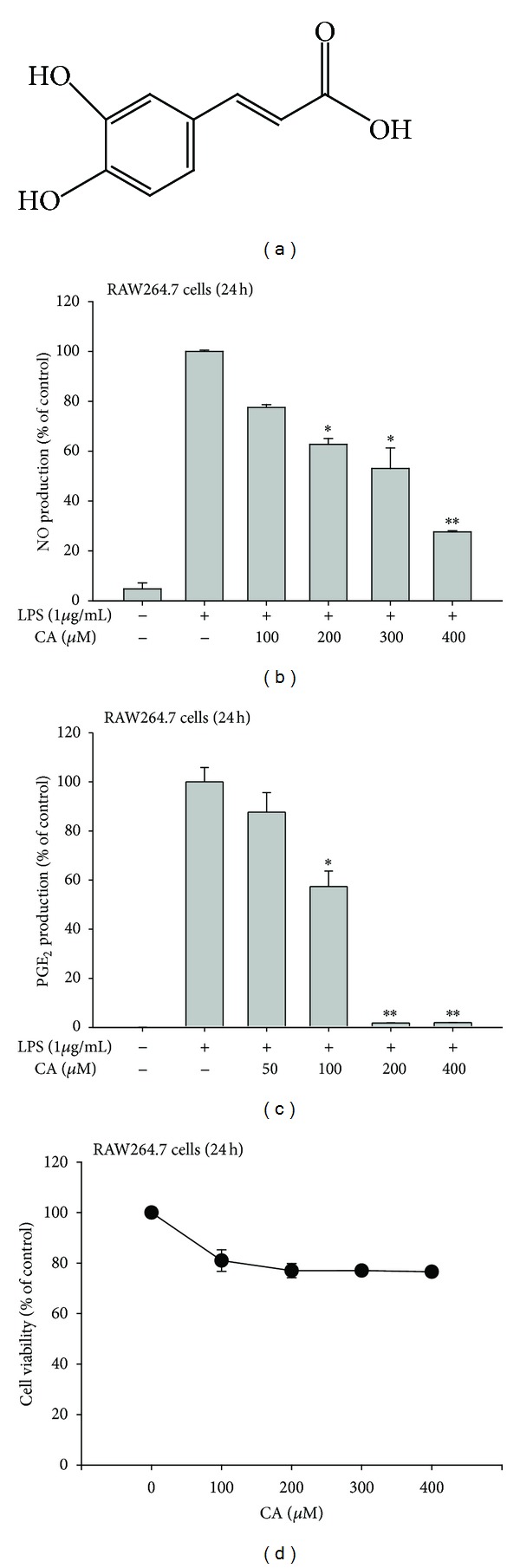
Effect of CA on NO and PGE_2_ production and viability in LPS-treated RAW264.7 cells. (a) The chemical structure of CA. ((b) and (c)) Levels of NO and PGE_2_ were determined by the Griess assay and EIA, respectively, from supernatants of RAW264.7 cells that had been treated with CA (0 to 400 *μ*M) in the presence or absence of LPS (1 *μ*g/mL) for 24 h. (d) RAW264.7 cell viability was determined by MTT assay. *P < 0.05 and **P < 0.01, compared to the control.

**Figure 2:**
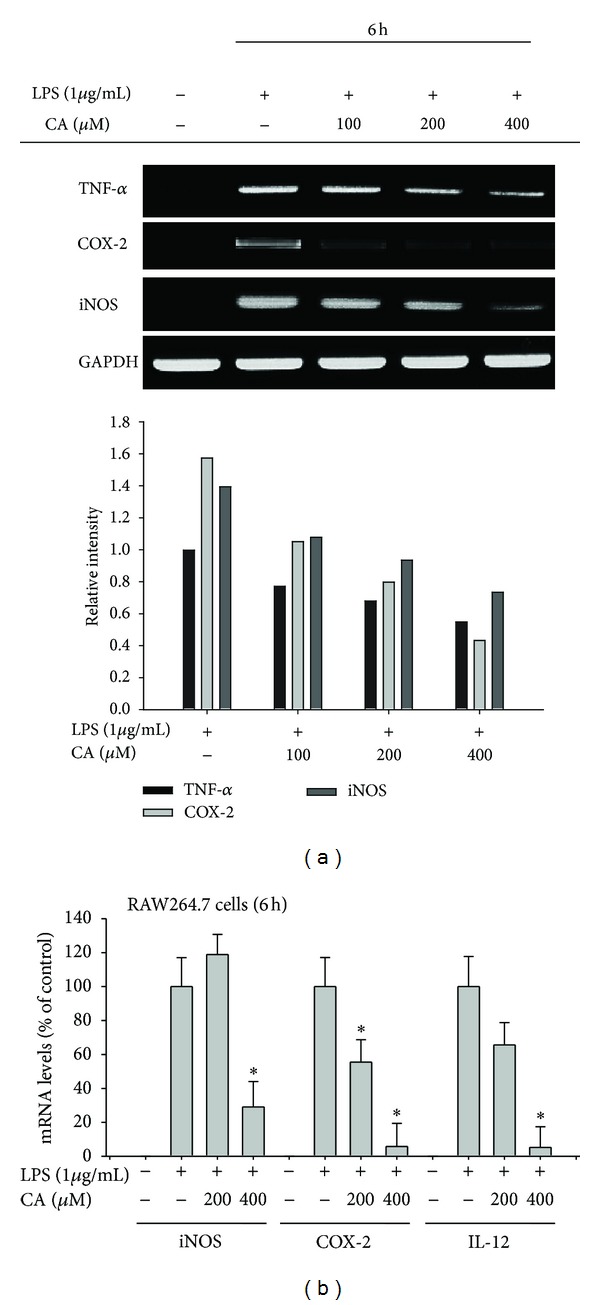
Effect of CA on the expression of proinflammatory gene mRNA. ((a) and (b)) mRNA levels of the genes encoding iNOS, TNF-*α*, IL-12, COX-2, and GAPDH were determined by semiquantitative or quantitative PCR. Relative intensity was calculated using GAPDH level by the DNR Bioimaging system. *P < 0.05, compared to the control.

**Figure 3:**
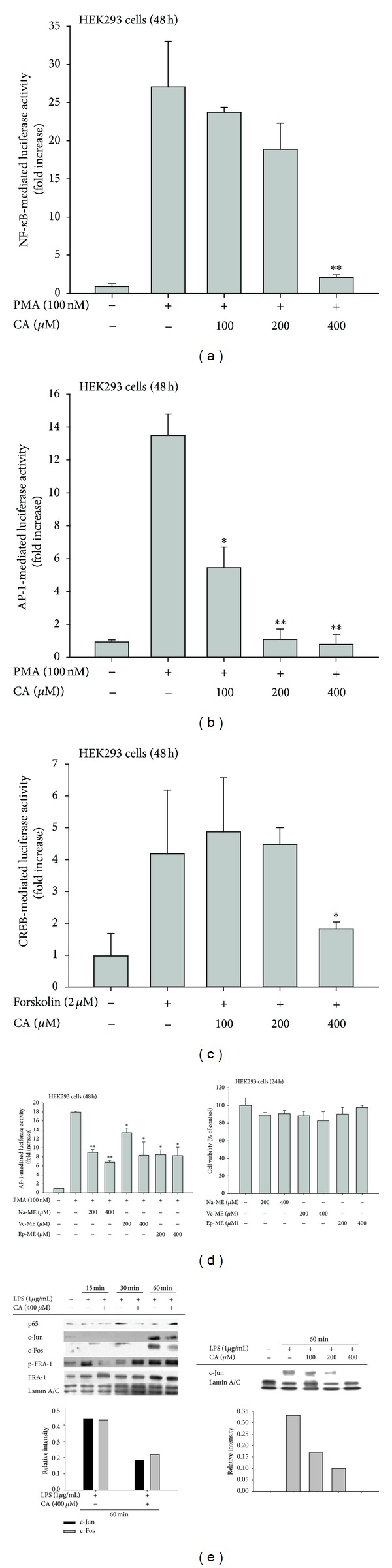
Effect of CA on transcription factor activation. ((a), (b), (c), and (d) left panel) HEK293 cells that had been cotransfected with NF-*κ*B-Luc, AP-1-Luc, or CREB-Luc plasmid constructs (1 *μ*g/mL each) and *β*-gal (transfection control) were treated with CA (0 to 400 *μ*M) or CA-containing plant extracts (Na-ME, Vc-ME, and Ep-ME (200 to 400 *μ*g/mL)) in the presence or absence of PMA (100 nM) or forskolin (2 *μ*M). Luciferase activity was measured with a luminometer. (d) HEK293 cell viability was determined by an MTT assay. (e) Levels of p65/NF-*κ*B and AP-1 family proteins (c-Jun, c-Fos, FRA-1, and p-FRA-1) in the nuclear fractions of LPS-treated RAW264.7 cells cultured in the presence or absence of CA (100 to 400 *μ*M) were determined by immunoblotting analyses with antibodies against the total proteins. Relative intensity was calculated using total levels by the DNR Bioimaging system. *P < 0.05 and **P < 0.01, compared to the control.

**Figure 4:**
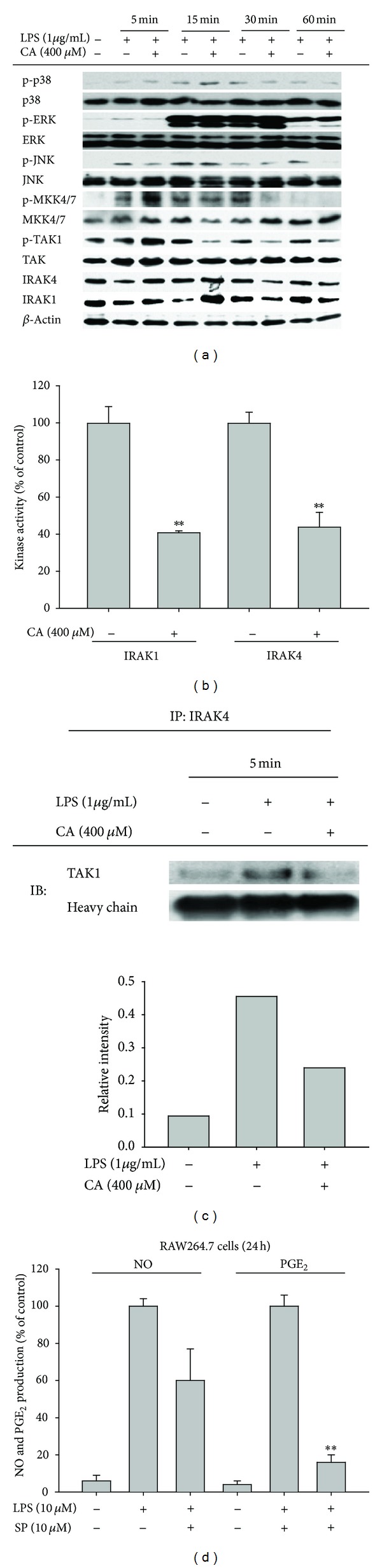
Effect of CA on the activation of AP-1 upstream signaling. (a) Levels of total or phosphoforms of MAPK (JNK, p38, and ERK) and JNK upstream enzymes (MKK4/7, TAK1, IRAK4, and IRAK1) from total lysates were determined by immunoblotting analyses with specific antibodies. (b) IRAK1 and IRAK4 kinase activities were determined by direct kinase assays with purified enzymes. The control for each enzyme (IRAK1 or IRAK4) was the activity obtained with vehicle treatment alone and was set at 100%. *P < 0.05 and **P < 0.01, compared to the control. (c) RAW264.7 cells (5 × 10^6^ cells/mL) were incubated with CA (400 *μ*M) in the presence or absence of LPS (1 *μ*g/mL) for 5 min. After preparing total lysates, the level of TAK1 binding to IRAK4 was identified by immunoprecipitation with an IRAK4 antibody and immunoblotting with antibodies against the rabbit immunoglobulin heavy chain and TAK1. (d) Culture supernatants prepared from LPS-treated RAW264.7 cells that were pretreated with a standard JNK inhibitor (SP600125 (SP)) were assayed for NO and PGE_2_. HC: heavy chain; **P < 0.01, compared to the control.

**Figure 5:**
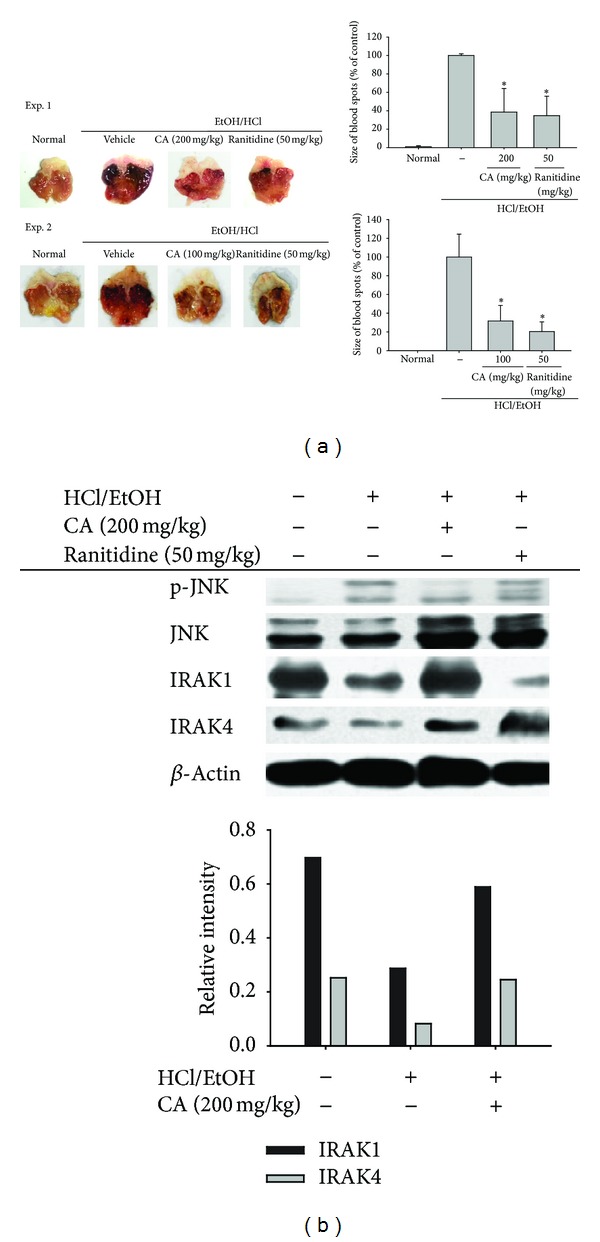
Effect of CA on inflammatory lesions in HCl/EtOH-treated stomachs in mice. (a) Mice orally administered either 200 mg/kg ((a) upper panel) or 100 mg/kg ((a) lower panel) or ranitidine (50 mg/kg) for 2 days were orally treated with HCl/EtOH. After 1 h, gastric lesions (the area (mm^2^)) in the stomach were measured with a pixel-counter; representative photos are shown. The gastritis index of the control group (inducer alone) was set to 100%. (b) Phosphoprotein or total protein levels of JNK, IRAK1, and IRAK4 in stomach lysates were determined by phosphospecific or total protein antibodies. Relative intensity was calculated using total levels by the DNR Bioimaging system. *P < 0.05, compared to control.

Data (Figures 1, 2(b), 3(a), 3(b), 3(c), 3(d), 4(b), 4(d), and 5(a)) are expressed as the mean ± standard deviation (SD) as calculated from one (*n* = 6) of two independent experiments. Other data are representative of three different experiments with similar results. For statistical comparisons, the results were analyzed by analysis of variance/Scheffe's post-hoc test and the Kruskal-Wallis/Mann-Whitney test. All *P* values <0.05 were considered statistically significant. All statistical tests were performed with SPSS (SPSS Inc., Chicago, IL, USA).

## 3. Results and Discussion

In this study, we found that CA (Figure 1(a)) can act as an effective anti-inflammatory drug. As such, this compound (100 to 400 *μ*M) effectively suppressed the LPS-mediated production of inflammatory mediators such as NO and PGE_2_ in macrophage-like RAW264.7 cells (Figures 1(b) and 1(c)). However, the cytotoxic activity of CA was marginal when compared to its inhibitory activity (Figure 1(d)), indicating that the action of CA was due to its specific immunopharmacological activity. Further analysis of inflammatory gene mRNA levels strongly suggested a potential inhibitory role for CA at the transcriptional level in LPS-treated inflammatory responses. Namely, the upregulated levels of TNF-*α*, COX-2, and iNOS were dose-dependently reduced by CA treatment (Figure 2(a)); this pattern was further confirmed by real-time PCR (Figure 2(b)), supporting the idea that the control of inflammatory gene mRNA levels might be a critical factor in CA-mediated anti-inflammatory activity. The *in vitro* anti-inflammatory activity of CA had been reported previously in monocytic THP-1 cells by measuring the levels of cytokines, including interleukin-1*β*, and adhesion molecule intercellular adhesion molecule-1 (ICAM-1) in response to PMA, influenza A, high-density lipoprotein, and TNF-*α* treatment [38–40]. Therefore, these reports and our data strongly support that CA is a flavonoid compound with stimulus-independent anti-inflammatory activity.

To determine how CA modulates inflammatory gene expression, we first employed a reporter gene assay with luciferase constructs that contained NF-*κ*B, AP-1, or CREB binding sites. Because PMA and forskolin were good inducers of NF-*κ*B-, AP-1-, and CREB-mediated luciferase activities, these inducers were used to treat CA-pretreated HEK293 cells. Interestingly, PMA-induced NF-*κ*B activation and forskolin-induced CREB activation were only inhibited at 400 *μ*M CA (Figures 3(a) and 3(c)), while AP-1 activation was strongly suppressed by CA, even at 100 *μ*M (Figure 3(b)). To confirm this effect, methanolic extracts known to contain CA were also tested for the suppression of AP-1-mediated luciferase activity. As expected, all of the extracts (Na-ME, Vc-ME, and Ep-ME) significantly inhibited AP-1 activation (Figure 3(d) left panel) without altering cell viability (Figure 3(d) right panel). Because the luciferase assay indicated possible transcription factor inhibition, we determined the nuclear translocation levels of the transcription factors. As Figure 3(e) (left) shows, the nuclear levels of p65, a subunit of NF-*κ*B, were reduced after a 30-min CA treatment, while the nuclear levels of AP-1 family members (c-Jun/c-fos and p-FRA-1) were suppressed by (100 to 400 *μ*M) at 60- and 15-min CA treatments, respectively (Figure 3(e) left and right panels). Therefore, these results indicate that AP-1 could be a strong target transcription factor for CA regulation.

To further understand the exact mechanism of CA-induced AP-1 inhibition, the signaling events upstream of AP-1 translocation were fully explored. We evaluated the phosphorylated or total levels of AP-1 upstream signaling enzymes, including MAPK and related upstream kinases [41], by immunoblotting analysis. As Figure 4(a) shows, CA did not suppress the phosphorylation of ERK and p38 but did inhibit the phosphorylation of JNK at 60 min without altering the total JNK levels. In agreement with this pattern, the phospholevels of the upstream kinases, including MKK4/7 and TAK1, were also clearly diminished at 30 and 15 min, respectively (Figure 4(a)). More interestingly, CA also blocked the degradation of IRAK1 and IRAK4, as assessed by total levels of these proteins, from 5 to 15 min (Figure 4(a)). Similarly, CA suppressed the enzymatic activities of IRAK1 and IRAK4 by as much as 60%, according to a direct kinase assay (Figure 4(b)). The CA-mediated inhibition of IRAK4 kinase activity was also observed in an immunoprecipitation analysis with anti-IRAK4 antibody and a subsequent immunoblotting analysis with anti-TAK1 (Figure 4(c)), indicating that the CA-mediated direct suppression of kinase activity affected the signaling complex formation between IRAK and TAK1. Furthermore, JNK was demonstrated to play a critical role in the regulation of the production of NO and PGE_2_, as shown in Figure 4(d); SP600125 (SP) reduced NO and PGE_2_ production by 40% and 85%, respectively (Figure 4(d)). The functional role of the JNK pathway in inflammatory responses has been previously reported. For example, cannabinoid receptor 2-mediated inflammatory signaling was shown to be controlled by JNK in human myeloid cells [42]. The oxidized phosphatidylcholine-induced regulation of TLR4-mediated inflammatory signaling was also reported to be JNK-dependent [43]. Furthermore, extracellular HSP60-induced inflammatory events were revealed to be controlled by JNK activation in cardiomyocytes [44]. Finally, the anti-inflammatory effects of numerous anti-inflammatory drugs and extracts such as inflexanin B, sargachromanol G, and artemisinin were confirmed to be mediated by direct or indirect JNK suppression [45–47]. Therefore, our results strongly suggest that a signaling cascade composed of IRAK1/4, TAK1, MKK4/7, and JNK, which are involved in TLR4-induced inflammatory responses, might be predominantly targeted by CA. Additionally, several enzymes such as Fyn, 5-lipoxygenase, matrix metalloproteinase, and ectonucleotidase are known to be pharmacological targets of CA and its derivate, CA phenethyl ester [17–20]; these findings imply that this compound might have a common structural unit that allows simultaneous interaction with various enzymes. The mechanism of action of this compound against a broad spectrum of pharmacological targets will be explored further in pharmacophore studies.

Ultimately, the application of CA as an orally administered therapy for *in vivo* inflammatory symptoms was tested. For this, we employed an acute inflammatory symptom model of HCl/EtOH-treated mice. Previously, we found that inflammation in this model was attenuated by celecoxib, a selective COX-2 inhibitor (data not shown), suggesting that COX-2 was involved in the specific inflammatory process. In addition to the inhibition of PGE_2_ production and COX-2 expression, CA also strongly ameliorated the gastritis symptoms at a level similar to that of the control drug ranitidine (Figure 5(a)). Intriguingly, analyses of JNK, IRAK1, and IRAK4 implied that these *in vitro* targets of CA could also be suppressed in *in vivo* inflammatory responses. Thus, JNK phosphorylation and IRAK1/4 degradation were suppressed by CA (Figure 5(b)). On the other hand, several other groups have demonstrated the *in vivo* activity of CA. The Yin group supplied CA in dietary pellets and found that 1-methyl-4-phenyl-1,2,3,6-tetrahydropyridine (MPTP) induced inflammatory brain injuries in mice were protected by CA via the suppression of NO and PGE_2_ production [48]. It was also confirmed that iron nitrilotriacetate (Fe-NTA)-induced renal inflammation was strongly attenuated by orally administered CA (20 and 40 mg/kg) [49]. The oral administration of CA (5–100 mg/kg) was shown to inhibit acetic acid-induced writhing and late-phase formalin-induced pain in mice at ED_50_ values of 22.38 and 10.92 mg/kg, respectively [50]. Therefore, previous reports and our results strongly suggest that CA is a good candidate compound for an orally administered *in vivo* inflammatory disease treatment.

**Figure 6:**
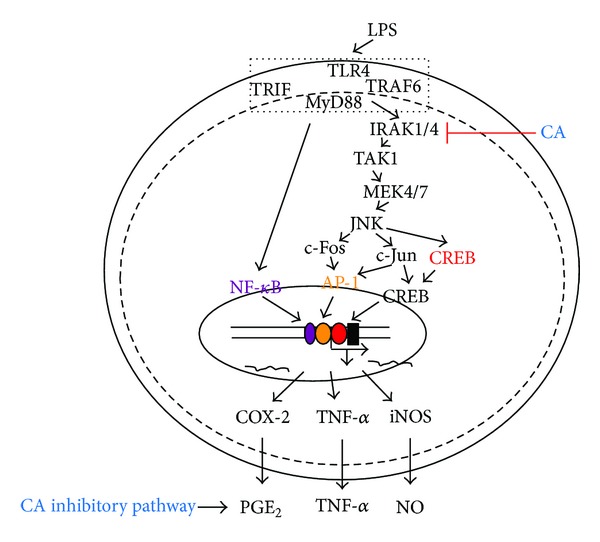
Putative CA-mediated inhibitory pathway of inflammatory signaling events.

In summary, we have confirmed that CA attenuates TLR4-mediated inflammatory responses such as NO and PGE_2_ production in LPS-treated RAW264.7 cells and ameliorates HCl/EtOH-induced acute gastritis symptoms. By analyzing the anti-inflammatory mechanisms, we found that CA strongly suppresses AP-1 activation via the suppression of related upstream signaling cascade enzymes, including IRAK1, IRAK4, TAK1, MKK4/7, and JNK, as summarized in Figure 6. In particular, a direct kinase assay strongly indicated that CA inhibits IRAK1 and IRAK4. Because these enzymes were recently revealed to play critical roles in various inflammatory responses, the ability of CA to suppress other IRAK1/4-mediated inflammatory symptoms will be evaluated further.

## Figures and Tables

**Table 1:** Primer sequences used in RT-PCR analysis.

Name		Sequence (5′ to 3′)
Real-time PCR		
iNOS	F	GGA GCC TTT AGA CCT CAA CAG A
	R	TGA ACG AGG AGG GTG GTG
COX-2	F	CAC TAC ATC CTG ACC CAC TT
	R	ATG CTC CTG CTT GAG TAT GT
TNF-*α*	F	TGC CTA TGT CTC AGC CTC TT
	R	GAG GCC ATT TGG GAA CTT CT
GAPDH	F	CAA TGA ATA CGG CTA CAG CAA C
	R	AGG GAG ATG CTC AGT GTT GG

Semiquantitative PCR		
iNOS	F	CCCTTCCGAAGTTTCTGGCAGCAG
	R	GGCTGTCAGAGCCTCGTGGCTTTGG
IL-12	F	GCGGGTCTGGTTTGATGA
	R	TGAACTGGCGTTGGAAGC
COX-2	F	CACTACATCCTGACCCACTT
	R	ATGCTCCTGCTTGAGTATGT
GAPDH	F	CACTCACGGCAAATTCAACGGCA
	R	GACTCCACGACATACTCAGCAC
